# Elucidating the clinical and immunological value of m6A regulator-mediated methylation modification patterns in adrenocortical carcinoma

**DOI:** 10.32604/or.2023.029414

**Published:** 2023-07-21

**Authors:** WENHAO XU, HAOMING LI, YASIR HAMEED, MOSTAFA A. ABDEL-MAKSOUD, SAEEDAH MUSAED ALMUTAIRI, AYMAN MUBARAK, MOHAMMED AUFY, WAEL ALTURAIKI, ABDULAZIZ J. ALSHALANI, AYMAN M. MAHMOUD, CHEN LI

**Affiliations:** 1 Department of Urology, Urological Surgery Research Institute, First Affiliated Hospital, Army Medical University (Third Military Medical University), Chongqing, China; 2 Department of Urology, Affiliated Hospital of Guilin Medical University, Guilin, China; 3 Department of Applied Biological Sciences, Tokyo University of Science, Tokyo, Japan; 4 Department of Botany and Microbiology, College of Science, King Saud University, Riyadh, Saudi Arabia; 5 Department of Pharmaceutical Sciences, Division of Pharmacology and Toxicology, University of Vienna, Vienna, Austria; 6 Department of Medical Laboratory Sciences, College of Applied Medical Sciences, King Saud University, Riyadh, Saudi Arabia; 7 Department of Life Sciences, Faculty of Science and Engineering, Manchester Metropolitan University, Manchester, UK; 8 Department of Biology, Chemistry, Pharmacy, Free University of Berlin, Berlin, Germany

**Keywords:** m6A, Tumor microenvironment, Adrenocortical carcinoma, Immunotherapy, Prognosis

## Abstract

N6-methyladenosine methylation (m6A) is a common type of epigenetic alteration that prominently affects the prognosis of tumor patients. However, it is unknown how the m6A regulator affects the tumor microenvironment (TME) cell infiltration in adrenocortical carcinoma (ACC) and how it affects the prognosis of ACC patients yet. The m6A alteration patterns of 112 ACC patients were evaluated, furthermore, the association with immune infiltration cell features was investigated. The unsupervised clustering method was applied to typify the m6A alteration patterns of ACC patients. The principal component analysis (PCA) technique was taken to create the m6A score to assess the alteration pattern in specific malignancies. We found two independent patterns of m6A alteration in ACC patients. The TME cell infiltration features were significantly in accordance with phenotypes of tumor immune-inflamed and immune desert in both patterns. The m6Ascore also served as an independent predictive factor in ACC patients. The somatic copy number variation (CNV) and patients prognosis can be predicted by m6A alteration patterns. Moreover, the ACC patients with high m6A scores had better overall survival (OS) and higher efficiency in immune checkpoint blockade therapy. Our work demonstrated the significance of m6A alteration to the ACC patients immunotherapy. The individual m6A alteration patterns analysis might contribute to ACC patients prognosis prediction and immunotherapy choice.

## Introduction

The unusual endocrine tumor generated from the adrenocortical is termed adrenocortical carcinoma (ACC). According to the Netherlands Cancer Registry, between 1993 and 2010, there were about 0.5 and 2.0 incidences of ACC per million individuals [1]. In addition to being uncommon, patients with ACC also have a poor 5-year overall survival (OS) rate of approximately 15% to 44% [2,3]. The prognosis of ACC is significantly affected by the disease stage upon diagnosis, with a 5-year survival rate of 80% for stage I and 13% for stage IV [4]. The sole therapy for early to mid-stage ACC is surgical operation, however, even after the tumor has been completely excised, generally 19%–34% local recurrence rate exists [5,6]. The advanced ACC patients who are unable to receive surgery or with metastases might benefit from other therapies, but it is achieved by oral chemotherapy-mitotane therapy currently. Therefore, understanding the m6A alteration method is essential for providing a novel strategy for ACC patients.

The m6A alteration, insertion of a methyl group to the N6 position of adenylic acid, was initially identified in the 1970s [7], since m6A alteration was shown to be dynamically reversible and obesity-associated protein was discovered to contain m6A demethylase function [8]. The methyltransferase complex, demethylase, and the related readers, which are referred to as “writers,” “erasers,” and “readers,” control the associated alteration of m6A. Numerous studies have interpreted the role of m6A methylation alteration in tumor invasion. In breast cancer, writers (METTL3) and erasers (ALKBH5) might promote tumor cell replication and migration by controlling the degree of m6A methylation [9]. However, the effect of m6A alteration in ACC patients is not well known yet.

Tumor microenvironment (TME), comprised of extracellular matrix, various soluble substances, immune cells, stromal cells, endothelium, and cancerous cells [10], mitigates tumor deterioration and affects the consequences of immunotherapeutic inhibitors in clinic [11,12]. The m6A alteration has been reported with close relation to immune cell infiltration that controls T cell homeostasis by targeting the IL-7/STAT5/SOCS pathways [13], and METTL3 drives M1 macrophage polarization by directly methylating STAT1 mRNA [14].

The noteworthy limitation of previous studies is merely a small amount of m6A regulators were analyzed, the systematic investigation is required, thus, in current work, we integrated various m6A alternations associated with TME cell infiltration features in ACC patients. ACC subjects from the TCGA and GEO databases were combined, and m6A alteration patterns were assessed. Based on the comparison of m6A alteration patterns and TME immune cell infiltration, two distinct m6A alteration patterns were identified, more importantly, these two patterns were very consistent with immune-inflamed and immune-desert phenotypes, respectively. This highlights that m6A alteration serves a vital role in the TME of ACC patients. Subsequently, the complicated biological processes were determined by enrichment analysis. Finally, m6A alteration was quantified by the created m6A score model which might develop a precise approach to enhance therapeutic utility.

## Materials and Methods

### Source and processing of the dataset for adrenocortical carcinoma

**Figure 1:**
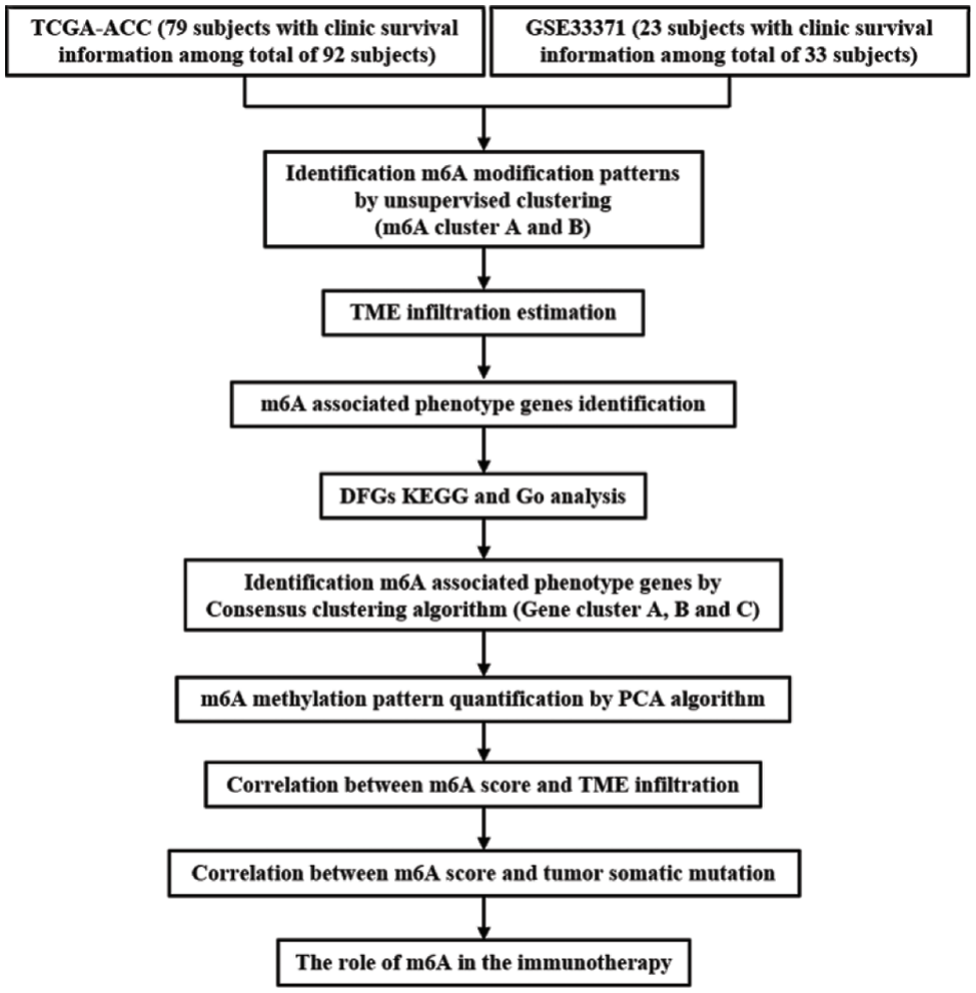
Overview of this work.

The workflow was shown in Fig. 1. In the current study, TCGA-ACC (79 subjects with clinic survival information among total of 92 subjects) and GSE33371 from the GEO database (23 subjects with clinic survival information among total of 33 subjects) were analyzed. As to datasets in TCGA, RNA sequencing data (FPKM value) of gene expression were downloaded from the Genomic Data Commons (GDC, https://portal.gdc.cancer.gov/) using the R package TCGAbiolinks, which was specifically developed for integrative analysis with GDC data. Then FPKM values were transformed into transcripts per kilobase million (TPM) values. Batch effects from non-biological technical biases were corrected using the “ComBat” algorithm of “sva” package. To further analyze copy number variation (CNV), we obtained the TCGA database’s basic nucleotide variation data. The GSE33371 microarray data from the Genetic Code U133 Plus 2.0 Array from Affymetrix. For microarray data from Affymetrix®, the raw “CEL” files were downloaded and adopted a robust multiarray averaging method with the affy and simplify packages to perform background adjustment and quantile normalization.

### Unsupervised clustering of 23 m6A regulators

23 well-known m6A regulators, including 13 readers, 8 writers, and 2 erasers were obtained from previous references [15]. The m6A-related genomic matrix from the combined sample according to 23 m6A regulators was extracted by ‘limma’ package. The unsupervised cluster analysis was applied to categorize the patients, we found several m6A alteration patterns based on the expression of 23 m6A regulators. The Euclidean distance was chosen as the clustering metric in the sample clustering, and the K-means clustering approach was used to accomplish the clustering goal.

### Gene set variation analysis (GSVA)

The comparison of the variations across biochemical functions according to m6A alteration patterns was performed by the unsupervised, nonparametric “GSVA” R package. Specifically, “c2.cp.kegg.v7.4.symbols.” gene set was examined. The difference is obvious when adjusted *p* < 0.05. To create the differential path heatmap, the first 20 differential pathways are chosen. The ‘limma’, ‘GSEABase’, ‘GSVA’, and ‘pheatmap’ packages, were taken to complete the above analyses.

### TME cell infiltration estimation

Single-sample gene set enrichment analysis algorithm (ssGSEA) was used to determine the relative abundance of each cell infiltrate in the sample, including activated B cells, CD56 Natural killer cells, eosinophil monocytes, and many others. A box graphic was used to illustrate the distinct immunological invading cells.

### DEG screening for m6A alteration patterns

The differentially expressed genes (DEGs) according to distinct m6A alteration patterns were determined by the empirical Bayesian, *p* < 0.001 was considered as significance. The cellular component, biological process, and molecular function of DEGs were examined by the Gene Ontology (GO) database. Additionally, the Kyoto Encyclopedia of Genes and Genomes (KEGG) database was used to conduct the pathway analysis of DEGs.

### The creation of the m6A gene signature

We developed a scoring method, termed as m6Ascore, to assess the m6A alteration pattern of individual ACC patient. The detailed steps were as follows. Cox regression analysis was utilized to identify DEGs with prognostic value, and then the unsupervised cluster was used to analyze different clusters with significant prognosis. Subsequently, m6A relevant gene signature was constructed by the principal component analysis (PCA), and both principal component 1 and 2 were selected. The advantage of this method is to focus on the score in the set with the largest block of well-correlated (or anti-correlated) genes, while down-weighting contributions from uncorrelated genes. Finally, the m6Ascore was defined by a method similar to Gene-gene interaction (GGI) [16,17]:

$$m6Ascore=\sum \left(PC1_{i}+PC2_{i}\right)$$

where, i stands for the expression of a gene associated to m6A.

### The function of the m6A score

The associations of m6Ascore and immune infiltrating cells, tumor mutation burden (TMB), m6A alteration pattern, m6A gene cluster type, and genes associated with immunotherapy were investigated to demonstrate the clinical value of m6Ascore, *p* < 0.05 was regarded as significant.

### Statistical analysis

The Kruskal-Wallis test was used for the nonparametric test, and one-way ANOVA was used for the parametric test. The association between m6Ascore and TMB was determined by Spearman correlation analysis. The survival was constructed by the Kaplan-Meier curve, the significance was evaluated by the log-rank test. The subjects were evaluated by PCA, paired with patient survival, and then separated into two groups using the optimal cutoff value given by the ‘Survminer’ R software program. The hazard ratios (HR) and independent prognostic variables of the m6A regulatory gene and m6A phenotypic associated gene were calculated by Cox regression. The ‘RCircos’ R software was used to create the position circle map of the m6A regulator CNV on the 23 chromosomes. The waterfall was created by “maftools” package, and the box diagram was created by the ‘ggpubr’ package. R 4.0.5 was used to do all analyses, and *p* < 0.05 was regarded as significant.

## Results

### The genomic landscape of *m6A* regulatory mutation in ACC

**Figure 2:**
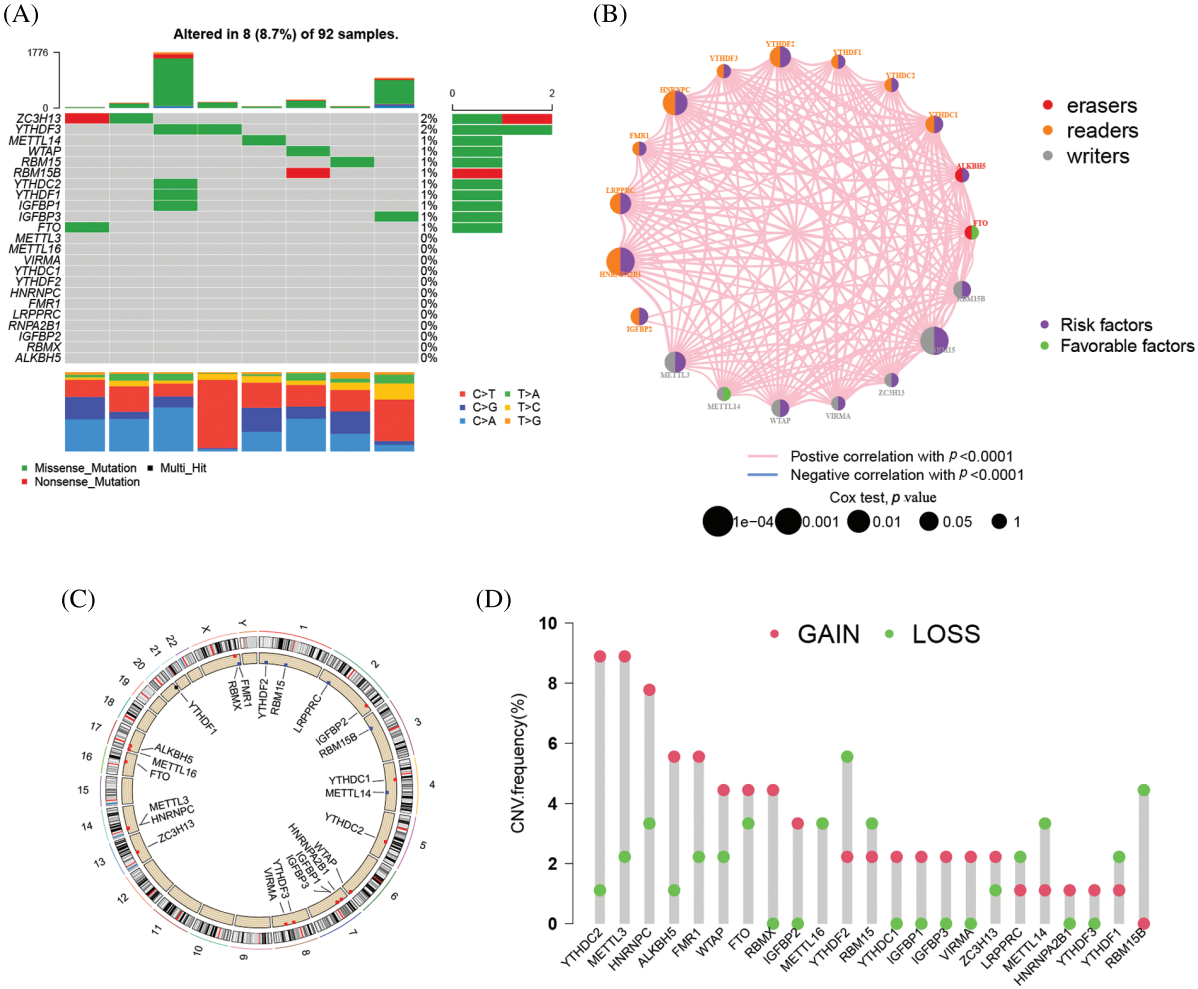
The mutation frequency of 23 m6A related genes in 92 patients with ACC. (A) Each column represents a single patient. The bar chart above showed TMB. The number on the right indicated the mutation frequency of each gene, and the bar chart on the right showed the proportion of each mutation type. The stacked barplot below showed the fraction of conversions in each sample. (B) Interactions between m6A regulators in adrenocortical carcinoma. The circle size represented the effect of each regulator on the prognosis, and the range of values calculated by Log-rank test was *****p < 1e−0.4, ****p < 0.001, ***p < 0.01, **p < 0.05 and *p < 1, respectively. Green on the right half of the dot, prognostic risk factor; purple on the right half of the dot, prognostic favorable factor. The lines linking the regulators showed their interactions, and thickness showed the correlation strength between regulators. Negative correlation was marked with blue and positive correlation with pink. The color of the left half of the dot represents the type of m6A regulator, writers, erasers and readers were marked in gray, red, and orange, respectively. (C) The location of CNV mutations in m6A regulators on 23 pairs of chromosomes according to the TCGA-ACC cohort. (D) CNV mutation frequencies of m6A regulators in the TCGA-ACC cohort. The height of the bars represents the frequency of mutation. Copy number deletions are green dots; copy number amplifications are red dots.

23 m6A regulators total, eight writers, two erasers, and thirteen readers, were used in this study. The m6A regulatory gene was altered in 8 of the 92 samples from TCGA cohort, yielding an 8.7% mutation frequency (Fig. 2A). The nonsense mutation is the second most frequent mutation after missense mutations. ZC3H13, YTHDF3, METTL14, WTAP, RMB15, RMB15B, YTHDC2, YTHDF1, IGFBP1, IGFBP3, and FTO were among the m6A regulatory genes with mutations. The univariate Cox regression analysis revealed 18 m6A regulators were related to the prognosis of ACC patients. The network node diagram showed the relationship between m6A regulators and prognosis of ACC patients (Fig. 2B and Suppl. Table S1). According to Fig. 2C, the CNV mutation of m6A regulator could be found on 23 pairs of chromosomes. Amplification accounted for the majority of CNV alterations of 23 m6A regulatory genes, while CNV deletions were in METTL14, METTL16, RMB15, RMB15B, LRPPRC, and YTHDF1 (Fig. 2D).

### 23 regulators mediate the patterns of m6A alteration

**Figure 3:**
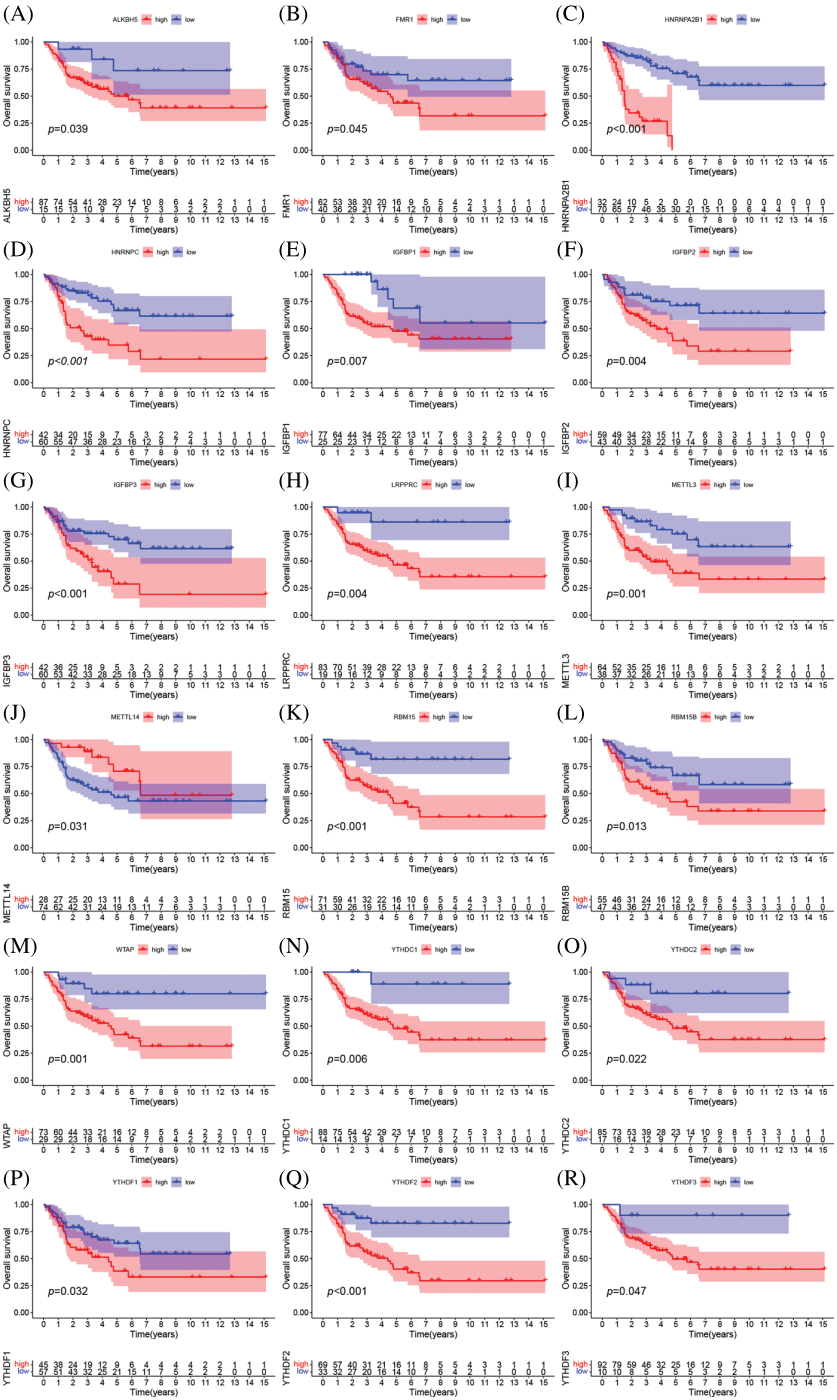
The prognostic analysis of ACC patients was based on the expression levels of m6A-related genes Kaplan-Meier curves for patients with high or low ALKBH5 (A), FMR1 (B), HNRNPA2B1 (C), IGFBP3 (D), LRPPRC (E), METTL3 (F), YTHDF3 (G), YTHDF2 (H), YTHDF1 (I), HNRNPC (J), IGFBP1 (K), IGFBP2 (L), METTL14 (M), RBM15 (N), RBM15B (O), YTHDC2 (P), YTHDC1 (Q), WTAP (R) expression in the merge cohort (TCGA-ACC and GSE33371).

**Figure 4:**
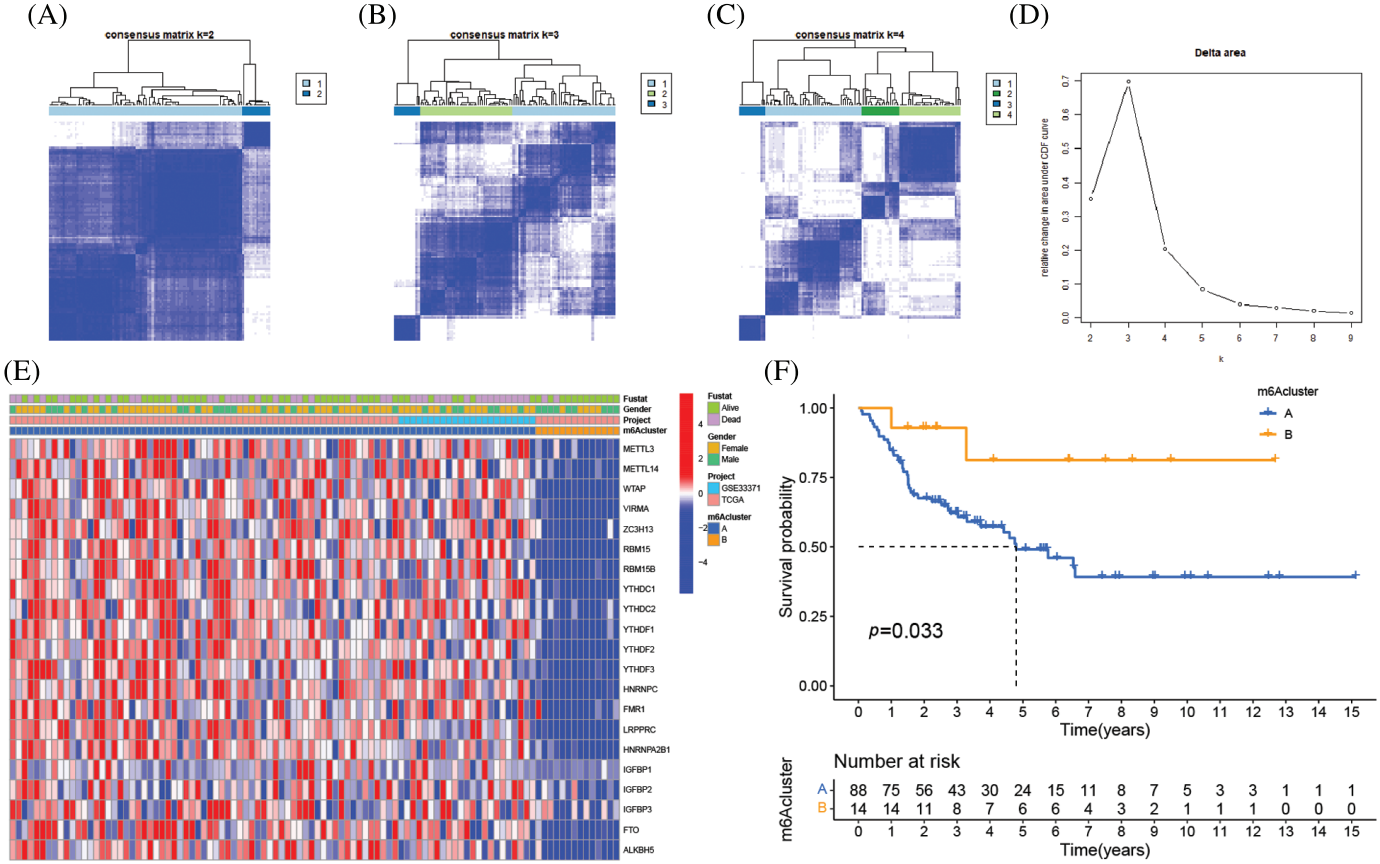
Molecular typing of ACC patients was based on m6A-related genes. (A–C) Consensus matrices of the merged cohort (TCGA-ACC and GSE33371) for k = 2–4. (D) The relative change in area under CDF curve for k = 2 to 9. (E) Unsupervised clustering of 23 m6A regulators in two independent adrenocortical carcinoma cohorts. The m6A clusters, gender, survival state and cohort names were used as patient annotations. Each column represented patients and each row represented m6A regulators. (F) Survival analyses for the two m6A modification patterns based on 112 patients with adrenocortical carcinoma from the merge cohort (TCGA-ACC and GSE33371) including 98 cases in m6A clusterA and 14 cases in m6A clusterB. Kaplan-Meier curves with Log-rank *p* value 0.033 showed a significant survival difference between the two m6A modification patterns. m6A clusterB showed significantly better overall survival than m6A cluster A.

The clinical characteristics of the subjects from TCGA-ACC and GSE33371 that were included in this work are shown in Suppl. Table S2. Based on the expression levels of m6A-related genes, the survival curves of ACC patients are shown in Fig. 3, there exists a strong association between writers, erasers, and readers as well as between the expression of the m6A regulator in the same functional category. Unsupervised cluster analysis was used to categorize, and the expression of 23 m6A regulators was used to distinguish between two distinct m6A alteration patterns, 98 Pattern A cases and 14 Pattern B. These two patterns are termed m6A cluster A and m6A cluster B. (Figs. 4A–4E and Suppl. Table S3). The prognostic study of the two types of m6A clusters revealed that m6A cluster B had a considerable survival advantage (Fig. 4F).

### TME cell infiltration variations under two m6A alteration patterns

**Figure 5:**
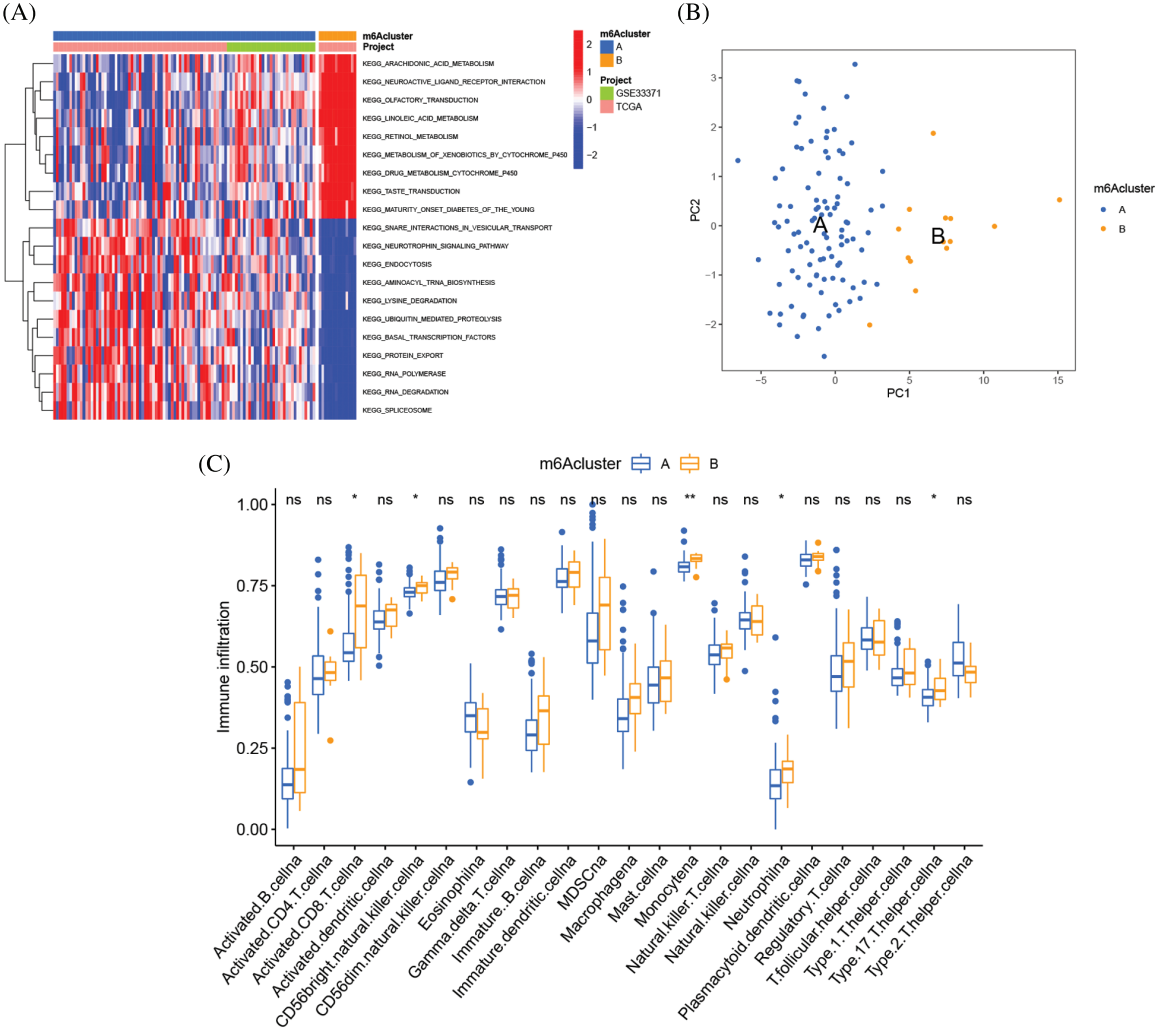
TME cell infiltration variations under two m6A alteration patterns. (A) GSVA enrichment analysis showing the activation states of biological pathways in distinct m6A modification patterns. The heatmap was used to visualize these biological processes, and red represented activated pathways and blue represented inhibited pathways. The adrenocortical carcinoma cohorts were used as sample annotations. (B) The abundance of each TME infiltrating cell in three m6A modification patterns. The upper and lower ends of the boxes represent an interquartile range of values. The lines in the boxes represented the median values, and black dots showed outliers. The asterisks represent the statistical *p* value (*p < 0.05; **p < 0.01). (C) Principal component analysis of the transcriptome profiles of two m6A modification patterns, showing a remarkable difference of transcriptome between different modification patterns.

GSVA enrichment analysis was applied to examine the biological differences between the two types of m6A clusters. The signal transduction pathways RNA degradation, aminoacyl tRNA biosynthesis, basal transcription factors, and neurotrophic signaling pathways were all considerably enriched in the m6A cluster A, as shown in Fig. 5A and Suppl. Table S4. The arachidonic acid metabolism, drug metabolism, cytochrome p450 metabolism, and linoleic acid metabolism pathways were all considerably concentrated in m6A cluster B. The m6A cluster B had much more immune cell infiltration than m6A cluster A, particularly activated CD8 T cells, CD56 bright NK cells, monocytes, neutrophils, and type 17 T helper cells (Fig. 5B). The immune-inflamed phenotype was defined by the presence of T cells that express CD4 and CD8 in the tumor parenchyma, in conjunction with myeloid cells and monocytes [18]. Additionally, individuals with the immune-inflamed phenotype have a better prognosis since they have better reaction to immunotherapy, which is supported by our findings. The transcriptome profiles of the m6A alteration pattern were then subjected to PCA, and the results revealed a substantial difference between the m6A cluster A and m6A cluster B in TME cell infiltration characterizations (Fig. 5C). Cluster B was designated as an immunological-inflamed phenotype, which is defined by immune activation and adaptive immune cell infiltration, whereas Cluster A was designated as an immune-desert phenotype, which is characterized by the suppression of immunity.

### Genetic function evaluation of DEGs and creation of m6A gene clusters

**Figure 6:**
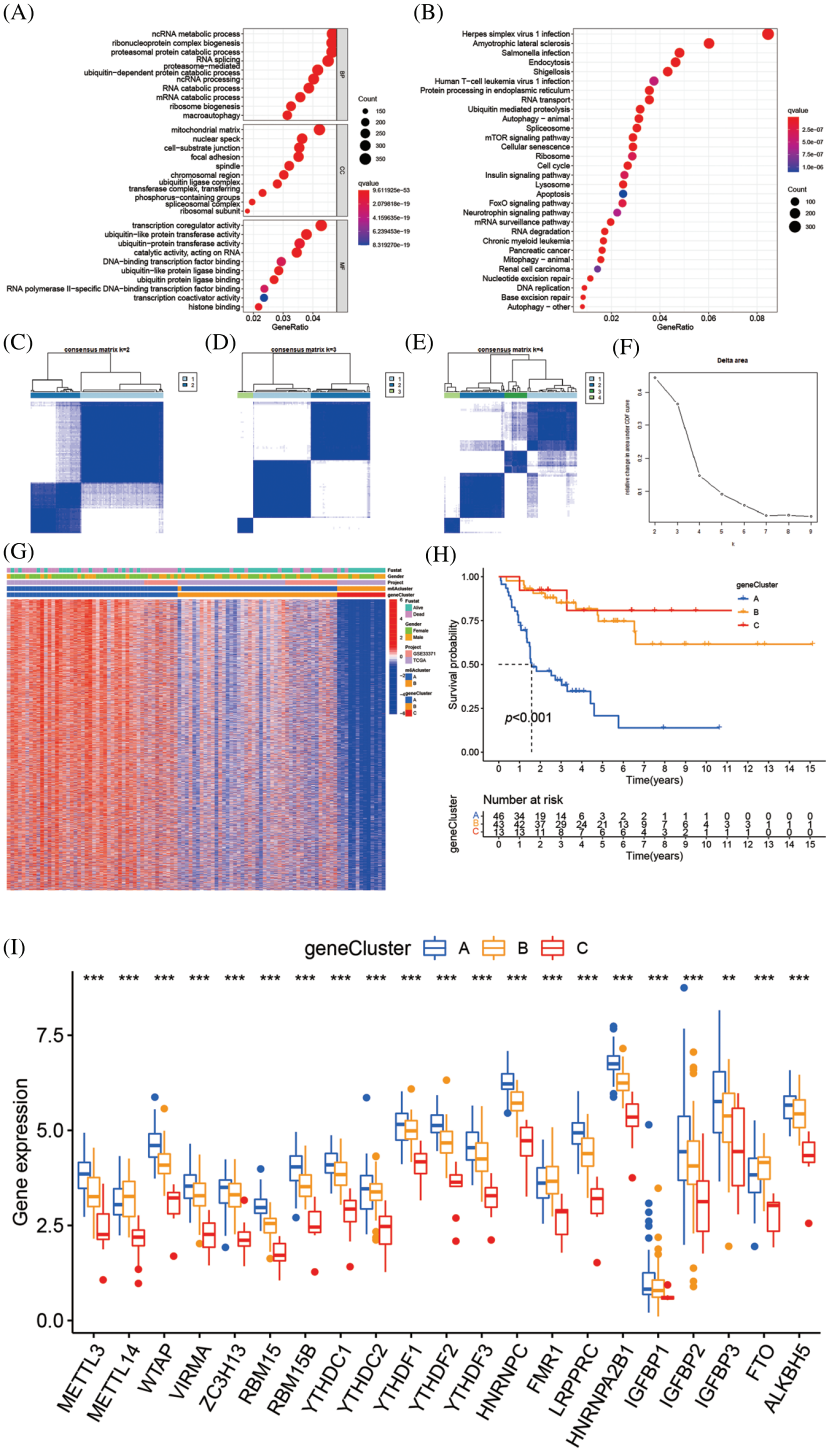
Genetic function evaluation of DEGs and creation of m6A gene clusters (A) The GO analysis of DEGs between m6A clusterA and m6A cluster B. (B) The KEGG analysis of DEGs between m6A cluster A and m6A cluster B. (C–E) Consensus matrices of consensus clustering of m6A DEGs for k = 2–4. (F) The relative change in area under CDF curve for k = 2 to 9. (G) Unsupervised cluster analysis on 8874 DEGs associated with m6A phenotypes. The m6A clusters, m6A gene cluster, gender, survival state and cohort names were used as patient annotations. (H) Survival analyses for the three m6A gene modification patterns based on 112 patients with adrenocortical carcinoma including 49 cases in m6A gene cluster A, 50 cases m6A gene cluster B and 13 cases in m6A cluster C. Kaplan-Meier curves with Log-rank *p* value < 0.001 showed a significant survival difference among the three m6A gene cluster. The m6A gene cluster C showed significantly better overall survival than the other m6A gene cluster. (I) The expression of 23 m6A regulators in three gene cluster. The upper and lower ends of the boxes represent an interquartile range of values. The lines in the boxes represented the median values, and black dots showed outliers. The asterisks represent the statistical *p* value (**p < 0.05; ***p < 0.01; ****p < 0.001). The one-way ANOVA test was used to test the statistical differences among three gene clusters.

Total of 8874 DEGs related to the m6A alteration pattern was identified by “limmma” package, followed by GO and KEGG enrichment (Suppl. Tables S5 and S6). The DEFs were enriched in the catabolic process of mRNA, the cell-substrate junction, and transcription coregulator activity biological processes, which demonstrate the non-negligible effect of m6A alteration in CC, BP, and MF regulation (Fig. 6A). The KEGG analysis of DEGs revealed variations in the mTOR signaling route, the insulin signaling pathway, the mRNA surveillance pathway, and other pathways (Fig. 6B). Further unsupervised cluster analysis of DEGs was conducted, the ACC patients were divided into three distinct genomic subtypes, dubbed m6A gene clusters A–C, as shown in Figs. 6C–6F. The majority of the follow-up for patients with m6A gene cluster A focused on mortality. The features of patients with m6A gene cluster B were comparable to those of patients with m6A gene cluster A. Contrarily, the majority of the follow-up for patients with m6A gene cluster C characteristics focused on live monitoring because they were almost all in the m6A cluster B pattern (Fig. 6G). The altered genomic phenotype of m6A was significantly correlated with the OS rate of ACC patients, according to the Kaplan-Meier curve. The prognosis for patients with m6A gene cluster A was the worst, whereas the prognosis for patients with m6A gene cluster B was the best (Fig. 6H). Among three m6A gene clusters, we found there was a significant difference 23 m6A regulators expressions. FTO, FMR1, and METTL14 were highly expressed in m6A gene cluster B, while the majority of the m6A-related genes were highly expressed in m6A gene cluster A (Fig. 6I).

### Correlation between m6A score and phenotypes associated with m6A

**Figure 7:**
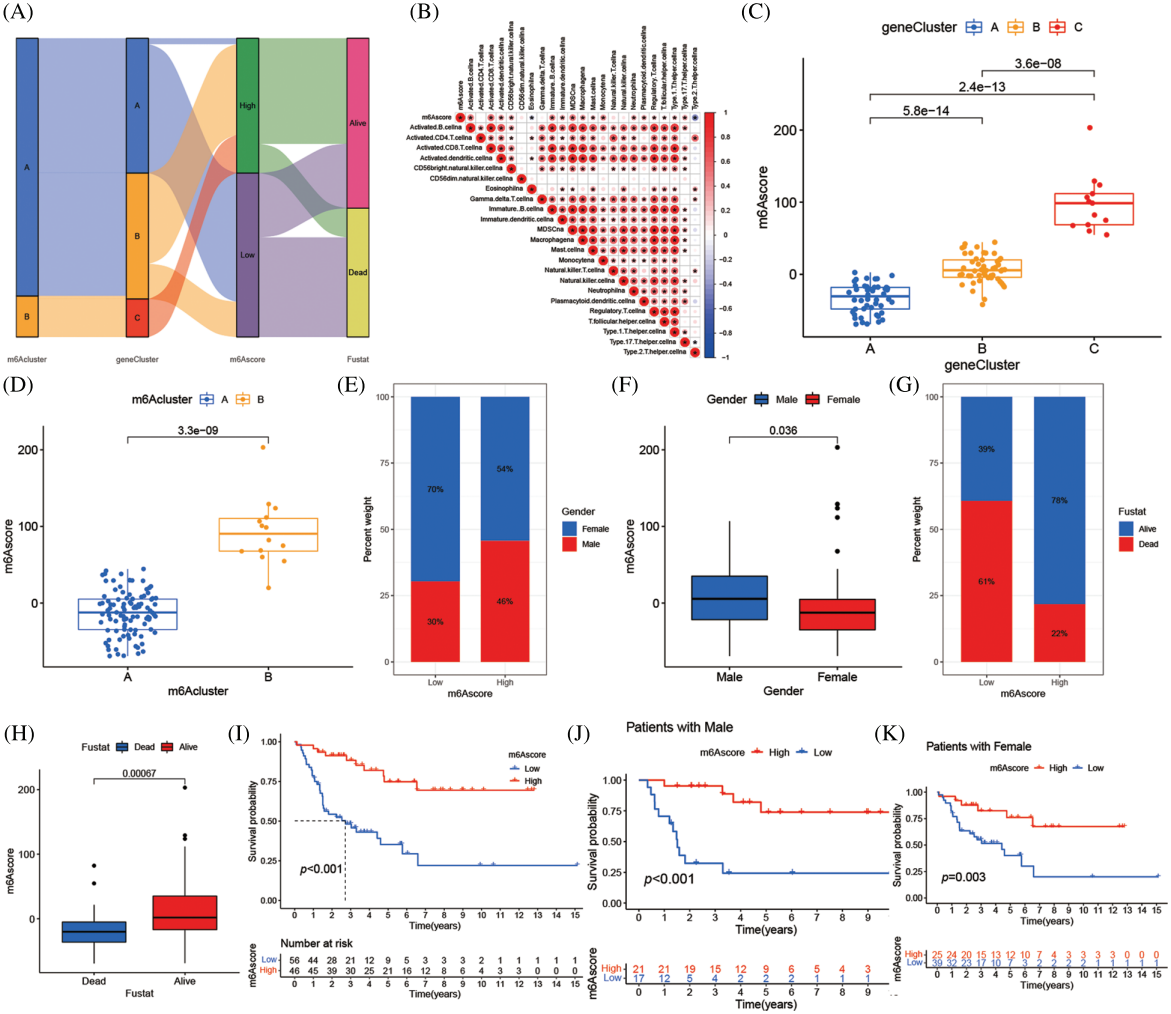
Correlation between m6Ascore and phenotypes associated with m6A. (A) Alluvial diagram showing the changes of m6A clusters, gene cluster, m6Ascore and survival state of patients with adrenocortical carcinoma. (B) Correlations between m6Ascore and immune infiltrating cells in the tumor microenvironment. Negative correlation was marked with blue and positive correlation with red. (C) Differences in m6Ascore among three gene clusters in the merge cohort (TCGA-ACC and GSE33371). The Kruskal Wallis test was used to compare the statistical difference between three gene clusters (****p < 0.001). (D) Differences in m6Ascore among two m6A modification patterns in merge cohort (TCGA-ACC and GSE33371). The Kruskal Wallis test was used to compare the statistical difference between three gene clusters (****p < 0.001). (E) The gender proportion of patients with low m6A score group and high m6A score group. Male/female: 30%/70% in the low m6Ascore group and 46%/54% in the high m6Ascore group. (F) There were differences in m6Ascore between different genders. (G) Proportion of survivors with low m6A score group and high m6A score group. Alive/Dead: 39%/61% in the low m6Ascore group and 78%/22% in the high m6Ascore group. (H) There were differences in m6Ascore between the different survival status of adrenocortical carcinoma patient. (I) Survival analyses for low (56 cases) and high (46 cases) m6Ascore patient groups in merge cohort (TCGA-ACC and GSE33371) using Kaplan-Meier curves (*****p < 0.0001, Log-rank test). (J) Survival analyses for low and high m6Ascore patient groups in patients with male. (K) Survival analyses for low and high m6Ascore patient groups in patients with females.

The m6A mutation pattern in ACC patients was determined by the created the m6Ascore system. The association between the m6A cluster, m6A gene cluster, m6Ascore, and the survival status of specific patients was shown in the Sankey diagram. The m6Ascore was created according to the m6A alteration pattern of DEGs in the PCA (Fig. 7A and Suppl. Table S7). Additionally, the association between m6Ascore immune cell infiltration in TME was investigated, and most immune cells, including activated B cells, active CD8 T cells, and type 17 T helper cells, were substantially associated with m6Ascore (Fig. 7B). In the m6A altered model with the high abundance of immune cell infiltration, the higher m6Ascore was associated with inflammation in the immune system, while the lower m6Ascore was associated with the immunological desert in the low abundance of immune cell infiltration model. The Kruskal-Wallis test revealed the median m6Ascore of m6A gene cluster B was higher than that of m6A gene cluster A. Additionally, there were notable variations in m6Ascore amongst m6A gene clusters (Fig. 7C). The three-stage grading of patients could be made by the m6Ascore, that grade the prognosis of patients more precisely. The median m6A score of the m6A cluster B was higher than m6A cluster A (Fig. 7D). When the population was divided by gender, the men subgroup showed higher m6Ascore than the women (Figs. 7E and 7F). The low m6Ascore subgroup had higher percentage of deaths (Figs. 7G and 7H). Additionally, the patient prognosis was accessed by m6Ascore, high m6Ascore patients had longer survival (Fig. 7I). Immune cell infiltration was more pronounced in patients with high m6Ascores which might lead to longer survival. This phenotype also existed in the population divided by gender (Figs. 7J and 7K).

### m6A alteration in tumor somatic mutation features

**Figure 8:**
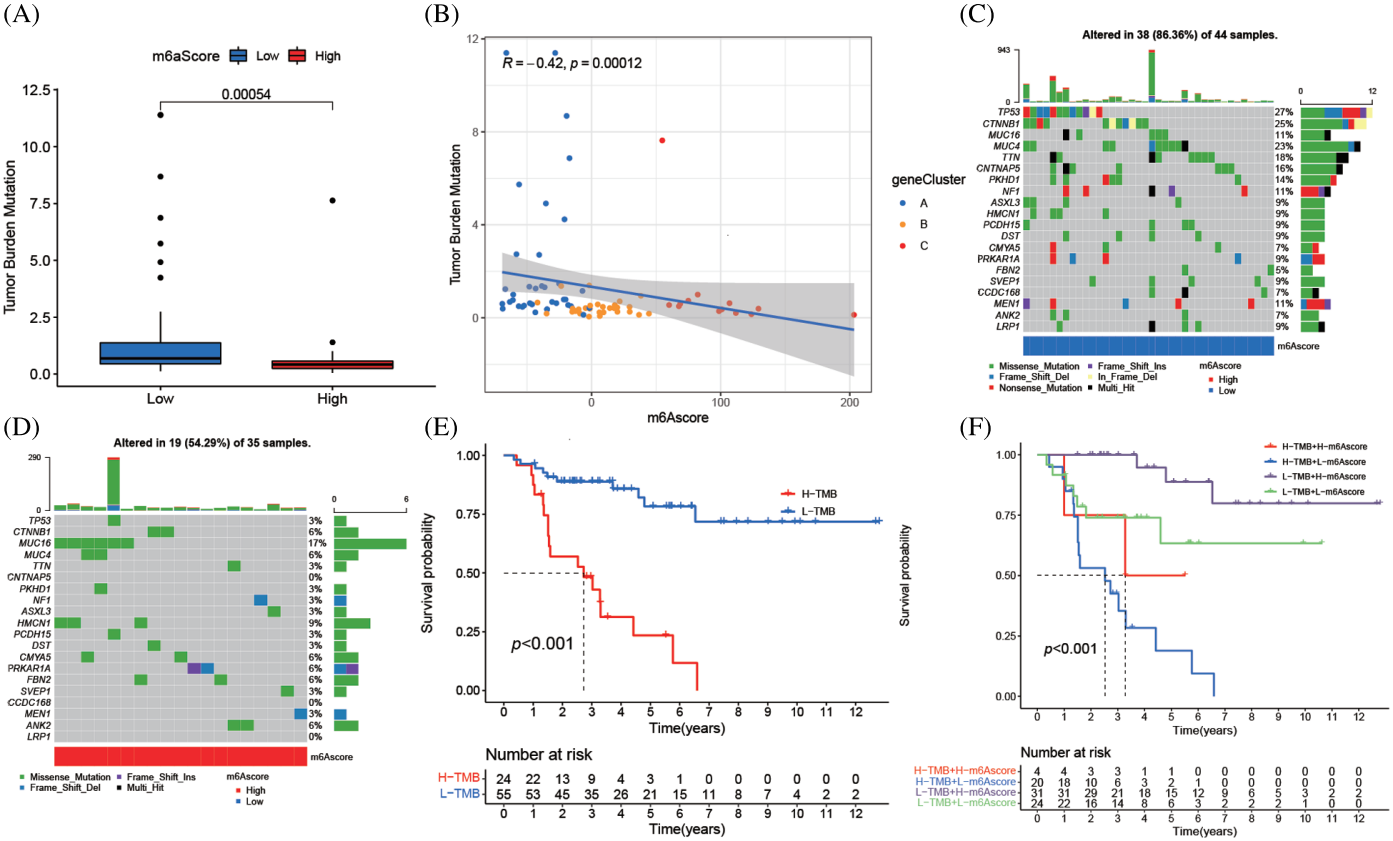
m6A alteration in tumor somatic mutation features. (A) The difference of tumor mutation burden between high m6Ascore and low m6Ascore (*****p < 0.0001). (B) The correlation between m6Ascore and tumor mutation burden. (C) The waterfall plot of tumor somatic mutation established by low m6Ascore. Each column represented individual patients. The upper barplot showed TMB, the number on the right indicated the mutation frequency in each gene. The right barplot showed the proportion of each variant type. (D) The waterfall plot of tumor somatic mutation established by high m6Ascore. Each column represented individual patients. The upper barplot showed TMB, the number on the right indicated the mutation frequency in each gene. The right barplot showed the proportion of each variant type. (E) Survival analyses for low and high tumor mutation burden patient groups in the merge cohort (TCGA-ACC and GSE33371) using Kaplan-Meier curves (*****p < 0.0001, Log-rank test). (F) Survival analyses for subgroup patients stratified by both m6Ascore and tumor mutation burden using Kaplan-Meier curves. H, high; L, Low; TMB, tumor mutation burden (*****p < 0.0001, Log-rank test).

The association between m6Ascore and tumor somatic mutations was assessed by “maftools” package, the substantial difference in TMB between the groups with high and low m6Ascores (Fig. 8A). Additional assay revealed the percentage of TMB decreased as the m6Ascore increased that a negative association between the m6Ascore and TMB (Fig. 8B). As shown in the waterfall chart (Fig. 8C), 38 cases altered among 44 samples (86.36%) in low m6Ascore group, and 19 cases altered among 35 samples (54.29%) in high m6Ascore group (Fig. 8D). ACC patients with low TMB have longer survival (Fig. 8E), furthermore, low TMB with high m6Ascore group has the best prognosis, while high TMB with low m6Ascore has the worst prognosis (Fig. 8F).

### m6A alteration pattern’s function in immunological checkpoints

**Figure 9:**
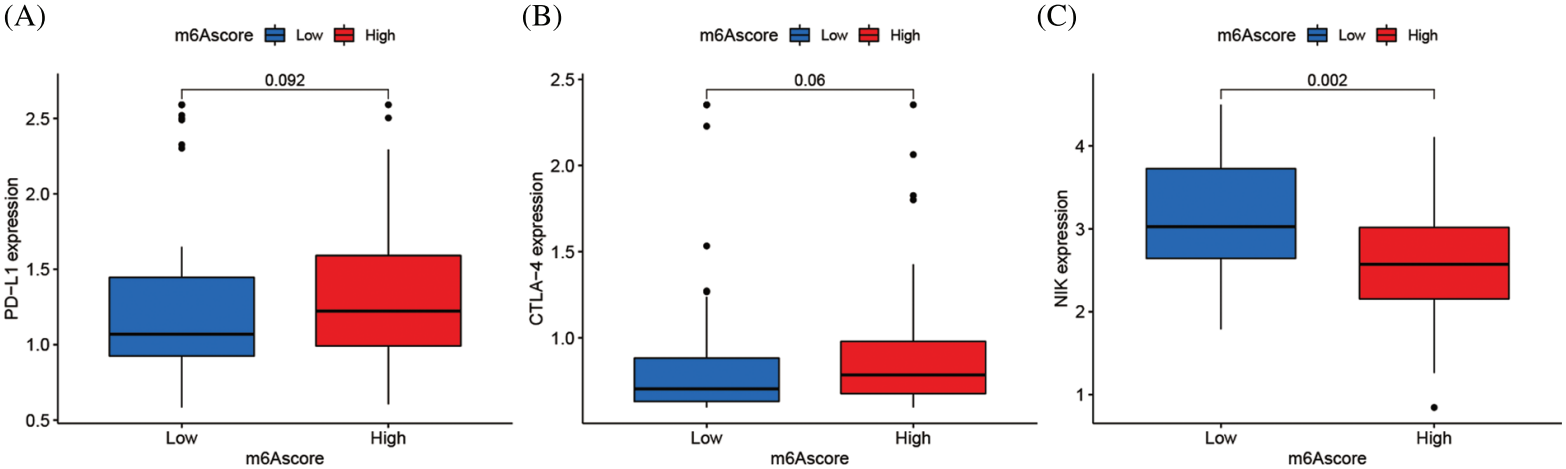
m6A alteration in immunological checkpoints. (A) Difference of PD-L1 gene expression between high and low m6Ascore. (B) Difference of CTLA-4 gene expression between high and low m6Ascore. (C) Difference of NIK gene expression between high and low m6Ascore.

Clinical immunotherapy, including PD-1/L1, CTLA4, and NIK blocker is crucial in malignancies management. The responses of groups with high and low m6Ascores to immune checkpoint blockade therapy were assessed in the merging cohort (TCGA-ACC and GSE33371). The ACC Patients with high m6Ascores had substantially increased levels of PD-L1 and CTLA4 expression (*p* < 0.01), suggesting a possible therapeutic response (Figs. 9A and 9B). However, the therapeutic potential of the NIK blocker might be weaker, since the high m6Ascore group had much lower NIK expression than the low m6Ascore group (Fig. 9C).

## Discussion

On RNA adenine, including mRNA and lncRNA, the methylation alteration known as m6A may occur [7]. Similar to another epigenetic control of DNA and histone alteration, the m6A alteration is dynamically reversible in mammalian cells [19]. The m6A RNA alteration affected RNA at many points during its life cycle, including RNA processing, nuclear export regulation, translation, and RNA degradation [20]. Evidence suggests that m6A alteration is intimately linked to the development of tumors. The expression level of m6A-related protein, a key regulator of tumor formation and progression, often defines the pathological course of the tumor [21]. m6A alteration has been shown to promote or inhibit cancer in several tumor types, including breast cancer [9], lung cancer [22], and acute myeloid leukemia [23], while preventing glioblastoma [24]. The impact of ACC m6A alteration mode on a patient’s prognosis is unclear since it is an uncommon endocrine malignant tumor. We combined the effects of 23 m6A regulators, in contrast to most studies that concentrated on one or two m6A regulators, to shed light on the function of two distinct m6A alteration patterns. This could help us to understand TME anti-tumor immune response clearly and develop efficient immunotherapy.

We integrated the m6A-related data from the ACC in the TCGA database and the GEO database, formed two clusters using unsupervised cluster analysis, and found that the two m6A clusters had significantly different OS and TME infiltration characteristics. Here, we identified two unique m6A methylation alteration patterns based on 23 m6A regulators. The TME cell invasion in these two patterns was noticeably different. Cluster B was defined by the activation of adaptive immunity, which corresponds to an immune-inflamed phenotype, whereas cluster A was characterized by the suppression of immunity, which corresponds to an immune-desert phenotype. The immune-desert phenotypes could be considered non-inflamed tumors. The immune-inflamed phenotype, referred to as a hot tumor, is characterized by a significant immune cell infiltration in the TME. In particular, m6A cluster B had greater immune cell infiltration than m6A cluster A, including activated CD8 T cells, CD56 NK cells, monocytes, neutrophils, and type 17 T helper cells. TME immune cell infiltration was widespread in ACC, according to our hypothesis, immune pathway activity was affected by m6A-related regulators, resulting in a range of cell infiltration characteristics. Through death receptor-mediated or mechanism-independent apoptosis, CD56 NK cells might promote the death of tumor cells [25]. The development of early immunological responses, adaptive responses (IFN-c), and the control of NK cells (IL-10) may be significantly influenced by CD56 bright NK cells [26]. T cell receptor-mediated cytotoxicity affects T cells and NK cells, moreover, the higher degree of T cell infiltration also improves protection against cancer [27]. Interestingly, the m6A cluster survival curve supports our hypothesis that patients with high levels of immune cell infiltration had a better prognosis. The immune cell infiltration of TME might prevent the emergence and progression of ACC, which offers evidence for immunotherapy utilization.

The prognostic variations across various m6A alteration patterns were strongly associated with immune-related biological pathways according to ssGSEA, GO, and KEGG analyses. DEGs, which were regarded as m6A-related characteristic genes, clustering outcome was comparable to the m6A cluster. The establishment of three gene clusters based on the m6A signature gene and their strong association with the OS of ACC patients further confirmed the link between m6A alteration and the progression of ACC. Therefore, analyzing the m6A alteration pattern will deepen the understanding of immune cell infiltration features in TMEs. The m6A alteration pattern of a single ACC patient was assessed by m6Ascore, considering the individual heterogeneity of m6A alteration. The higher m6Ascore, correlated with an immune-inflamed phenotype, was present in the m6A altered model that was characterized by high abundance immune cell infiltration. The lower m6Ascore in the m6A altered mode was associated with immunological desert phenotype and low quantity immune cell infiltration. Further investigation revealed that ACC patients with high m6Ascores had longer OS, demonstrating the predictive value of the m6A alteration, and the inhibitory effect of high immune cell infiltration in TME. In summary, the m6Ascore model could be applied to predict the TME infiltration features and ACC patients survival accurately.

TMB is a detectable biomarker [28] that can be used to identify patients with good responses to immune checkpoint inhibitor therapy [29]. The higher TMB has also been shown to correlate with clinical benefits from ICI therapy within multiple tumors, in our study, TMB is also a novel antigens generation that activates anti-tumor immunity [30–32]. These immunotherapies have shown remarkable anti-tumor effects in prior clinical trials [27,33], and have received approval for a wide range of tumor types with a variety of indications, including metastatic castration-resistant prostate cancer [34]. Our findings revealed a substantial inverse relationship between m6Ascore and TMB, despite ACC patients with high TMB having a poorer OS, it is an immunoreactive tumor and a “hot tumor,” which has significant therapeutic implications since these patients might be more sensitive to immune medicine.

We also verified that there was a difference in NIK and PD-L1 expression between the two m6Ascore groups. Numerous costimulatory substances have an effect on NIK downstream proteins. According to a melanoma study, the loss of NIK resulted in both an increase in tumor burden and a reduction in tumor-infiltrating T cells, indicating that NIK is essential for both T cell survival and anti-tumor immunity. NIK-targeted therapy offers a perspective for investigating immunotherapy, as it identifies a novel regulatory component of T cell metabolism. The NIK-targeted treatment in combination with immune checkpoint inhibitors could be considered for ACC patients who missed the best chance for surgery. There are limitations in the current study, and bench experiments will be conducted to support our findings.

As for the validation study, the Kaplan-Meier curve and Log-rank test of low and high m6Ascore patients in the combined cohort to validate the accuracy of our model. Although these validated data are not from the same source as the data we modeled, in multiple datasets, m6Ascore has a credible prognostic value, demonstrating the reliability of our conclusions.

## Conclusions

In conclusion, TME immune cell infiltration features and m6A methylation alteration pattern could be assessed by the m6Ascore model. Furthermore, it was also applied to determine the TMB, OS, and m6A genotyping in ACC patients. More importantly, the effectiveness of novel immune checkpoint blockades (PD-1/L1 & CTLA4) based on the m6Ascore in the clinic was predicted, such as NIK-targeted treatment with immune checkpoint inhibitors, offering some new insights for ACC immunotherapy.

## Supplementary Materials

Table S1 Spearman correlation analysis of the 23 m6A modification regulators

Table S2 Basic information of datasets included in this study for identifying distinct m6A methylation modification patterns

Table S3 Estimating relative abundance of tumor microenvironment cells in ACC patients by the Single-Sample Gene-Set Enrichment Analysis (ssGSEA)

Table S4 The activation states of biological pathways by GSVA enrichment analysis

Table S5 Prognostic analysis of m6A phenotype-related genes using a univariate Cox regression model

Table S6 Functional annotation for m6A phenotype-related genes (Gene Ontology-Biological process)

Table S7 The changes of m6A clusters, survival time and m6Ascore

## Data Availability

All data used in this work can be acquired from the TCGA database (https://portal.gdc.cancer.gov/), GEO database (https://www.ncbi.nlm.nih.gov/geo/).
